# Amyloid β Induces Early Changes in the Ribosomal Machinery, Cytoskeletal Organization and Oxidative Phosphorylation in Retinal Photoreceptor Cells

**DOI:** 10.3389/fnmol.2019.00024

**Published:** 2019-02-22

**Authors:** Liting Deng, Kanishka Pushpitha, Chitra Joseph, Veer Gupta, Rashi Rajput, Nitin Chitranshi, Yogita Dheer, Ardeshir Amirkhani, Karthik Kamath, Dana Pascovici, Jemma X. Wu, Ghasem Hosseini Salekdeh, Paul A. Haynes, Stuart L. Graham, Vivek K. Gupta, Mehdi Mirzaei

**Affiliations:** ^1^Department of Molecular Sciences, Faculty of Science and Engineering, Macquarie University, Sydney, NSW, Australia; ^2^Faculty of Medicine and Health Sciences, Macquarie University, Sydney, NSW, Australia; ^3^School of Medicine, Deakin University, Waurn Ponds, VIC, Australia; ^4^Australian Proteome Analysis Facility (APAF), Macquarie University, Sydney, NSW, Australia; ^5^Cell Science Research Center, Department of Molecular Systems Biology, Royan Institute for Stem Cell Biology and Technology, Academic Center for Education, Culture and Research (ACECR), Tehran, Iran

**Keywords:** proteomics, TMT, Alzheimer’s disease, photoreceptor, autophagy, amyloid, retina

## Abstract

Amyloid β (Aβ) accumulation and its aggregation is characteristic molecular feature of the development of Alzheimer’s disease (AD). More recently, Aβ has been suggested to be associated with retinal pathology associated with AD, glaucoma and drusen deposits in age related macular degeneration (AMD). In this study, we investigated the proteins and biochemical networks that are affected by Aβ in the 661 W photoreceptor cells in culture. Time and dose dependent effects of Aβ on the photoreceptor cells were determined utilizing tandem mass tag (TMT) labeling-based quantitative mass-spectrometric approach. Bioinformatic analysis of the data revealed concentration and time dependent effects of the Aβ peptide stimulation on various key biochemical pathways that might be involved in mediating the toxicity effects of the peptide. We identified increased Tau phosphorylation, GSK3β dysregulation and reduced cell viability in cells treated with Aβ in a dose and time dependent manner. This study has delineated for the first-time molecular networks in photoreceptor cells that are impacted early upon Aβ treatment and contrasted the findings with a longer-term treatment effect. Proteins associated with ribosomal machinery homeostasis, mitochondrial function and cytoskeletal organization were affected in the initial stages of Aβ exposure, which may provide key insights into AD effects on the photoreceptors and specific molecular changes induced by Aβ peptide.

## Introduction

Amyloid beta (Aβ) is a highly toxic and hallmark aggregate-prone peptide associated with Alzheimer’s disease (AD) pathology. The disease affects around 5.7 million people in USA alone and is predicated to rise and affect over 13.8 million people by the middle of the century in USA (2018; Alzheimer’s Association, 2018). Recent studies indicate that the retina which is a neural offshoot of the brain, undergoes pathological changes during AD pathogenesis (Gupta et al., 2016a,b; Golzan et al., 2017). Increasing lines of evidence have shown that specific AD like pathology (development of amyloid plaques and neurofibrillary tangles) in the brain can also be found in the retina (Koronyo-Hamaoui et al., 2011a). Particularly, Aβ accumulation has been reported in the retinas of human AD subjects (Goldstein et al., 2003; Koronyo-Hamaoui et al., 2011b; Koronyo et al., 2017). The deposition of Aβ, derived from abnormal processing of amyloid precursor protein (APP) has also been found in the retinas of AD transgenic mice (Kin Chiu, 2013; Gupta et al., 2016b). Furthermore, Aβ accumulation and increased APP reactivity has been identified in other neurodegenerative disorders of the retina such as in glaucoma as well as in the drusen deposits in age related macular degeneration (AMD; Anderson et al., 2004; Gupta et al., 2014; Zhao et al., 2015). These findings highlight the common features shared by AD, AMD, and glaucoma in the context of Aβ amyloidosis. AMD is associated with the loss of photoreceptor neurons and anti-Aβ therapy has been shown to be protective and prevent vision loss (Ding et al., 2011). Aβ neutralization in the retina using specific antibodies was also shown to impart protection in AD animal models and in experimental glaucoma implicating its negative impact on the retinal neurons (Guo et al., 2007; Ding et al., 2008; Liu et al., 2009). In contrast, Aβ administration either through the subretinal injections or intravitreal space exhibits neural toxic properties (Guo et al., 2007; Liu et al., 2015).

The abnormal processing of Aβ and its oligomerization leads to formation of Aβ assemblies and activation of apoptotic pathways that are detrimental to the cell functioning and induce synaptic degeneration (Benilova et al., 2012). Other studies associate the toxicity of the Aβ peptides linked to inflammation and retinal degeneration (Ning et al., 2008; Liu et al., 2013). Retinal abnormalities such as sensory deficiencies, visual impairment and functional deficits have been reported in early AD (Berisha et al., 2007; Cheung et al., 2015; Golzan et al., 2017; Gangoda et al., 2018).

Despite emerging evidence on involvement of Aβ in retinal abnormalities, the precise mechanisms involved in Aβ accumulation and its subsequent effects on the retinal cells have remained ill-defined. To delineate the global level proteome expression changes induced by Aβ on the photoreceptor cell in culture, we, used tandem mass tag (TMT) quantitative proteomics, to assess the time and dose dependent cytotoxic effects of Aβ 1–42 peptide. Cultured neuronal cells have the capacity to internalize Aβ 1–42 from the culture media and exhibit toxicity effects such as ROS production within lysosomes and impairment of lysosomal membrane proton gradient leading to cell death (Ditaranto et al., 2001). Of note, an increasing body of evidence highlights that toxic effects of Aβ are mediated through oxidative stress, impairment of cytoskeletal organization and mitochondrial dysfunction which are implicated in AD, but have also been reported in AMD and glaucoma (Ratnayaka et al., 2015; Gupta et al., 2016a; Masuzzo et al., 2016). Our results establish the toxic effects of Aβ on photoreceptor cells and suggest that Aβ may specifically perturb biochemical pathways associated with protein synthesis, RNA processing, oxidative phosphorylation and cytoskeleton organization in a time and concentration dependent manner.

## Materials and Methods

### Cell Culture and Treatments

Mouse retinal cells (661 W cells) were grown in DMEM medium containing 10% (v/v) fetal bovine serum (FBS), 1% (v/v) penicillin/streptomycin (Thermofisher), in presence of 5% CO_2_ at 37°C incubator for 24 h, and then transferred into new plates to culture for another 24 h in the incubator (Gupta et al., 2015). Aβ 1–42 fragment (Sigma) was freshly prepared each time by dissolving in PBS and added into the culture plates to a final concentration of 5μm or 25μm for either 6 h or 24 h at 37°C as reported previously (Hansson Petersen et al., 2008). pTau Ser202/Thr205 (Mn1020, 1:1,000; Thermo Fisher), Tau (Tau46 40,191:1,000), pGSK3β Ser9 (93,361:1,000), GSK3β (93,151:1,000; Cell Signaling Technology), Anti-beta Actin [(AC-15) ab6276, 1:10,000] (Abcam). Horseradish peroxidase conjugated anti-rabbit and anti-mouse secondary antibodies (R&D Systems) were used to visualize the bands. Cells were separately harvested after treatments with Aβ for 6 and 24 h. Therefore, there were four separate sets of samples from four separate treatments consisting of two concentrations and two time-points: treatment 1 (T1_5 μm_6 h), treatment 2 (T2_5 μm_24 h), treatment 3 (T3_25 μm_6 h), and treatment 4 (T4_25 μm_24 h). Four biological replicates were prepared for each specific treatment, along with five control replicates. Cells were stored at −80°C until protein extraction.

### Protein Sample Preparation and Western Blotting

Cells were lysed in 200 μL ice-cold lysis buffer (50 mM Tris-HCl, pH7.5, 150 mM NaCl, 1%NP40, 1 mM EDTA, 0.1% SDS) containing protease inhibitor and sonicated using a probe sonicator (40 HZ × 2 pulses × 15 s) with 30 s interval on ice. Supernatant was transferred to a new tube and insoluble debris was removed by centrifugation at 13,000 rpm for 15 min at 4°C. Extracted proteins were reduced with 20 mM dithiothreitol (DTT) for 15 min at room temperature, and then alkylated with 50 mM iodoacetamide for 30 min in the dark at room temperature. The alkylation reaction was then quenched with additional 40 mM DTT for 15 min in the dark. Methanol/chloroform was applied to precipitate proteins and remove interfering detergents and other contaminants (Wessel and Flugge, 1984). The protein pellet was left to dry and then resuspended with 200 μL of 8 M urea in 100 mM Tris-HCl (pH = 8.5). Protein concentration was measured with bicinchoninic acid (BCA) assay kit (Pierce, Rockford, LI, USA) with bovine serum albumin as standards. The proteins (200 μg) were digested using trypsin (Promega, Madison, WI, USA) with a 50:1 protein: enzyme ratio and incubated at 37°C overnight. Digested peptides were then acidified with trifluoroacetic acid (TFA) to a final concentration of 1% (pH 2–3) and desalted using SDB-RPS (3 M-Empore) Stage-tips. Samples were eluted from Stage-tip using 200 μL of 80% acetonitrile/5% ammonium hydroxide. Dried peptides were dissolved in 100 mM HEPES (pH = 8.0) and peptide concentration was measured using Micro BCA assay kit (Pierce, Rockford, LI, USA). For western blotting, 30 μg of protein was separated using 10% SDS-PAGE and transferred to PVDF membrane for blotting. 5% skimmed milk buffer in Tris-HCL buffer containing saline was used to block the western blotting membrane. The primary antibody was added to the blot and incubated for the night at 4°C. This was followed by thoroughly washing the blots with Tris buffer saline (three times) and subsequently incubating the blots with horseradish peroxidase conjugated secondary antibody. The blots were extensively washed again, and images taken using the western blotting densitometric imager. The blots were developed using chemiluminescent substrate within the linear range of detection and data quantified and plotted using Graph pad prism as described previously (Gupta et al., 2010, 2012, 2017).

### TMT Labeling

**Figure 1:**
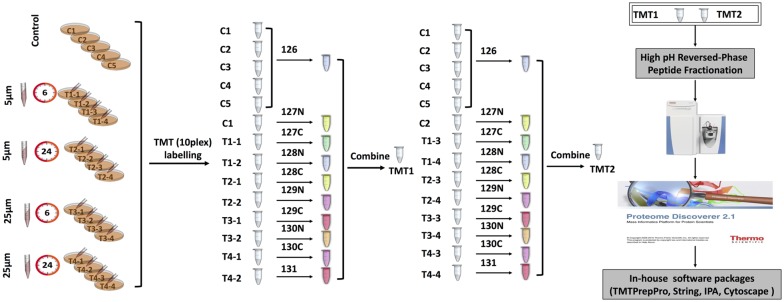
Tandem mass tag (TMT) labeling strategies and experimental workflow.

One-hundred microgarm of peptides from each sample were used for TMT labeling (Thermofisher) with 0.8 mg of labeling reagents per reaction. Labeling was carried out at room temperature for 1 h with occasional vortexing. To quench any remaining unbound TMT reagents, 8 μL of 5% fresh hydroxylamine was added, followed by vortexing and incubation at room temperature for 15 min. To accommodate 21 samples (five control replicates and 16 samples from four treatments), two separate 10-plex TMT experiments were carried out (Figure 1). For each TMT experiment, respective 10 labeled samples were combined and dried in a vacuum centrifuge. Peptides were reconstituted in 1% formic acid and desalted using Sep-Pak C18 cartridges (Waters, Milford, MA, USA). After high pH reversed-phase peptide fractionation, peptides were consolidated into 16 fractions. Dried fractions were resuspended in 1% formic acid, and then desalted again using SDB-RPS (3 M-Empore) stage tips (Chitranshi et al., 2018).

### LC-MS/MS Analysis

Fractionated and dried peptides were reconstituted in 40 μL of 0.1% formic acid and analyzed on a Q Exactive Orbitrap mass spectrometer (Thermo Scientific, San Jose, CA, USA) coupled to an EASY-nLC1000 nanoflow HPLC system (Thermo Scientific, San Jose, CA, USA). Reversed-phase chromatographic separation was performed on an in-house packed reverse–phase column (75 μm × 10 cm with Halo^®^ 2.7 μm 160 Å  ES-C18 (Advanced Materials Technology). Labeled peptides were separated for 2 h using a gradient of 1%–30% solvent B (99.9% acetonitrile/0.1% formic acid) and Solvent A (97.9% water/2% acetonitrile/0.1% formic acid). The Q Exactive mass spectrometer was operated in the data-dependent acquisition (DDA) mode to automatically switch between full MS and MS/MS acquisition. Following the Full MS scan spectra (from m/z 350–1,850), were acquired at resolution of 70,000 at m/z 400 and an automatic gain control) target value of 1 × 10^6^ ions. The top 10 most abundant ions were selected with precursor isolation width of 0.7 m/z for higher-energy collisional dissociation (HCD) fragmentation. HCD normalized collision energy was set to 35% and fragmentation ions were detected in the Orbitrap at a resolution of 70,000. Target ions that had been selected for MS/MS were dynamically excluded for 90 s.

### Database Searching

Raw data files were processed in Proteome Discoverer V2.1 (Thermo Scientific, San Jose, CA, USA) using search engines Mascot (Matrix Science, WI, UK). Data were matched against the reviewed SwissProt Mus musculus protein database (16,953 sequences, Feb 2018). The MS1 tolerance was set to ± 10 ppm and the MS/MS tolerance to 0.02 Da. Carbamidomethyl (C) was set as static modification, while TMT 10-plex (N-term, K), Oxidation (M), Deamidation (N, Q), Glu->pyro-Glu (N-term E), Gln->pyro-Glu (N-term Q) and Acetylation (Protein N-Terminus) were set as dynamic modifications. Percolator algorithm was used to discriminate correct from incorrect peptide-spectrum matches, and calculate statistics including q-value (FDR) and posterior error probabilities. Search results were further filtered to retain protein, peptide and PSM with FDR of <1% and only master proteins assigned *via* protein grouping algorithm were retained. Relative quantitation of proteins was achieved by pairwise comparison of TMT reporter ion signal to noise (S/N) ratios (in case of availability across all channel if not intensities are used), for example, the ratio S/N of the labels for each of treatment replicates (numerator) vs. the labels of their corresponding control replicates (denominator).

### Analysis of Differentially Expressed Proteins

Proteins were further analyzed using the in house developed TMTPrepPro analysis pipeline (Mirzaei et al., 2017b). The TMTPrepPro scripts are implemented in the R programming language and are available as an R package, which is accessed in our group through a graphical user interface provided *via* a local GenePattern server. All protein ratios with respect to the pooled control reference (label-126) were extracted and combined across runs. Two separate approaches were considered for the data analysis: the analysis of variance (ANOVA) analysis of all four treatments, and the pairwise comparisons of each treatment to the control. Differentially expressed proteins based on ANOVA comparison of log-transformed ratios were identified and clustered to check the conditions of Aβ peptide treatment and controls were well separated. For the pairwise comparisons, relative quantitation of protein abundance in Aβ peptide treatments compared to control was derived from the ratio of the TMT label S/N detected in each treatment to control, and differentially expressed proteins were identified based on student *t*-tests between control and treatment ratios. The overall fold changes were calculated as geometric means of the respective ratios. Differential expression required proteins to meet both a ratio fold change [>1.20 (up-regulated) or <0.833 (down-regulated)] and a *p*-value cut-off (*t*-test *p*-value < 0.05; Margolin et al., 2009; Kammers et al., 2015). The identified differentially expressed proteins through the pairwise comparisons were subjected to pathways enrichment analysis using Ingenuity Pathway Analysis (IPA)^1^; core analyses for each comparison were followed up with a comparison analysis. Identified proteins were mapped to genes using Ingenuity Pathway Knowledge Base (IPKB). Significant interaction networks (*p* < 0.05) and molecular and cellular functions were identified based on known protein–protein interactions in the Ingenuity knowledgebase. Networks naming is based on the most common functional group(s) present. Canonical pathway analysis is used to identify the function-specific genes amongst the up and down-regulated proteins. In addition, as a separate orthogonal approach, the differentially expressed proteins identified with ANOVA analysis were further classified according to pathways and biological processes using the Cytoscape stringApp plugin installed^2^. Significantly changed proteins were loaded into Cystoscope, and the Mus Musculus database in the StringDB was selected to reveal the protein interactions in the context of enriched pathways.

### MTT Assay for Cell Viability

661 W cells were grown in culture plates until around 85% confluency was achieved. The cells were then subjected to MTT (Sigma) treatment (0.5 mg/mL) in plates treated with different concentration of Aβ at indicated time-points and incubated at 37°C for 3 h as previously described (Chitranshi et al., 2017). The cells were lysed gently shaken and absorbance taken at 570 nm followed by plotting the results.

## Results

### Differential Effects of Aβ on the Proteome Profile of Photoreceptors Cell Line

**Figure 2:**
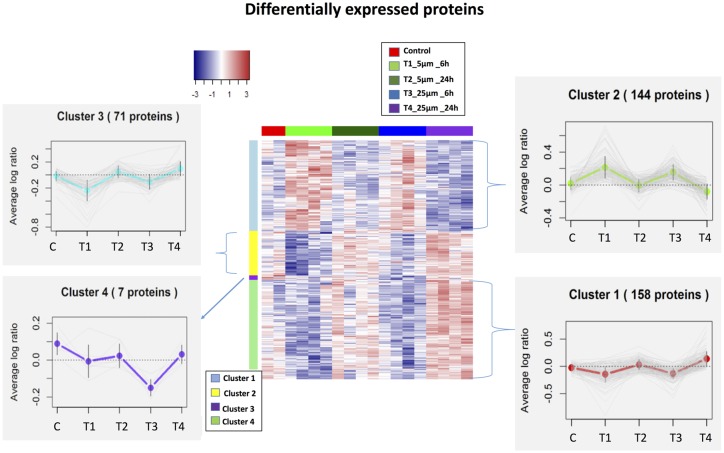
Heatmaps (hierarchical clustering) of the log-transformed ratios of differentially expressed proteins [differences between all experimental conditions *via* analysis of variance (ANOVA)] after Amyloid β (Aβ) treatments; row clustering only. Column colors indicate treatment type and the cluster patterns are detailed on the side plots.

We identified 5,837 proteins from 661 W photoreceptor cells [treated with various concentrations (5 and 25 μM) of Aβ at both 6 and 24 h], which were quantified by multiple peptides at an initial protein FDR of less than 1% (Supplementary Table S1). There were 380 proteins identified as differentially expressed between all treatments *via* an ANOVA (*p*-value <0.05, maximum absolute value of fold change ≥1.2). Hierarchical clustering analysis of proteins with differential abundance (380 proteins based on ANOVA analysis) illustrated the overall consistency of up or down regulation within 6 h treatments and 24 h treatments (Figure 2 and Supplementary Table S2). The proteome profile was relatively similarly affected when cells were treated for 6 h with either 5 or 25 μM Aβ concentrations. A similar distribution pattern of the differentially expressed proteins was also observed amongst the cells that were treated for 24 h with either 5 or 25 μM Aβ concentrations. This suggested that duration of treatment with Aβ was a key factor in regulating proteome changes in the 661 W photoreceptor cells.

**Figure 3:**
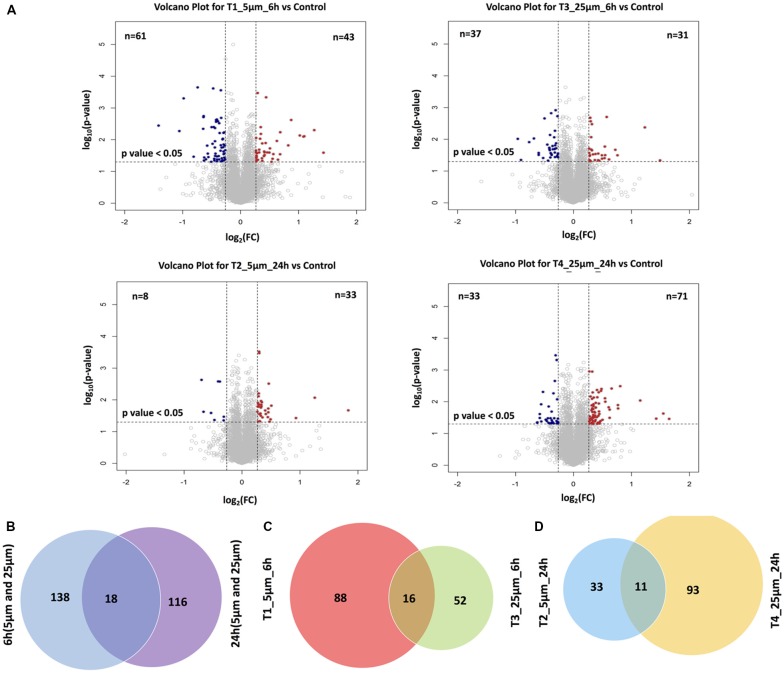
**(A)** Volcano plots demonstrating the dual thresholds for differentially regulated proteins. Proteins within the upper and outer quadrants meet both the fold change and *p*-value cut-off and are therefore considered as differentially regulated. **(B)** Venn diagram indicating the overlap between the differentially expressed proteins identified and quantified in cells after treated for 6 h and 24 h, respectively (1% FDR). **(C)** Venn diagram indicating the overlap between the differentially expressed proteins identified and quantified in T1 and T3 (1% FDR). **(D)** Venn diagram indicating the overlap between the differentially expressed proteins identified and quantified in T2 and T4 (1% FDR).

**Figure 4:**
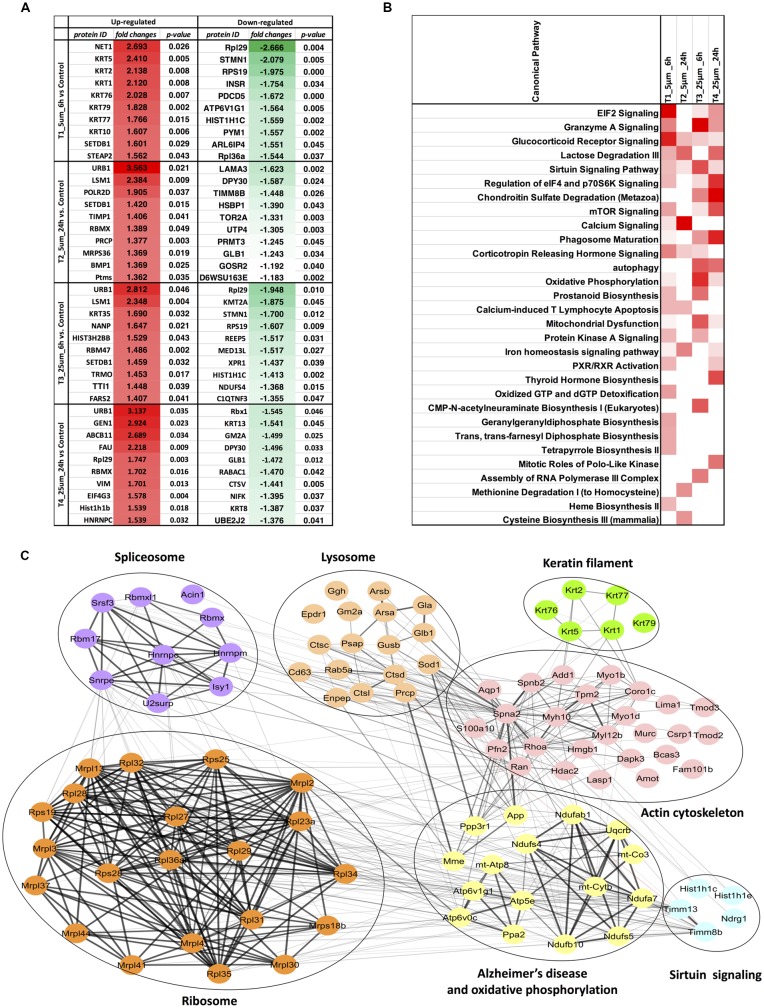
**(A)** Top 10 regulated proteins in four treatments. **(B)** Comparison of the top canonical pathways enriched from ingenuity pathway analysis (IPA) analysis of differentially regulated proteins (treatment vs. control) in four treatments. The significance of functional enrichment is highlighted with red color. **(C)** Functional interaction networks analyzed by the String Cytoscape plugin. One-hundred and one differentially expressed proteins were in pathways related to Alzheimer’s disease (AD). Network nodes are labeled with gene symbols.

For the four pairwise comparisons, quantified proteins observed to be significantly regulated in treatments *t*-test, *p*-value ≤0.05 and relative change from control at least ±20% (≥1.2 or ≤0.83-fold change) are shown in Figure 3A and Supplementary Table S3. A comparison between the control and experimental groups indicated 61 and 37 proteins, respectively downregulated upon treatment with 5 and 25 μM concentrations of Aβ at 6 h. In contrast, 43 and 31 proteins were respectively upregulated at these two concentrations at this time point. For the 24 h treatment group, we observed 8 and 33 proteins respectively downregulated while 33 and 71 proteins were up-regulated at 5 μM and 25 μM treatment groups. The highest number of down-regulated proteins was identified in the 5 μM treatment group at 6 h and the greatest number of up-regulated proteins was identified in the cells treated with 25 μM concentration for 24 h. Venn diagram plots further indicated that almost equal number of proteins was differentially expressed between the 6 and 24 h time-points (combined changes at 5 and 25 μM). However, when the data was plotted separately with respect to different Aβ treatment concentrations keeping the time constant, a higher number of differentially expressed proteins were identified at 5 μM (6 h) and 25 μM (24 h) concentrations compared to the other two groups. Interestingly, the number of differentially expressed proteins across various Venn plots remained largely similar (Figures 3B–D). The top 10 differentially regulated proteins (3.5 to −1.3-fold change) in the two Aβ treatment groups (5 and 25 μM) at 6 and 24 h time-points are listed (Figure 4A). Nucleolar pre-ribosomal-associated protein 1 (URB1) and U6 snRNA-associated LSM1 proteins were consistently upregulated at both 5 and 25 μM treatment groups at 24 h. RNA binding motif containing proteins such as RBMX and RBM47 were also up-regulated. Interestingly, ribosomal proteins such as Rpl29, RPS19, Rpl36a were downregulated in the photoreceptor cells treated with Aβ for 6 h. In contrast, histone methyltransferase complex regulatory subunit DPY30 expression was suppressed specifically in the groups treated with Aβ for 24 h.

### Pathway Classification of the Differentially Expressed Proteins

**Figure 5:**
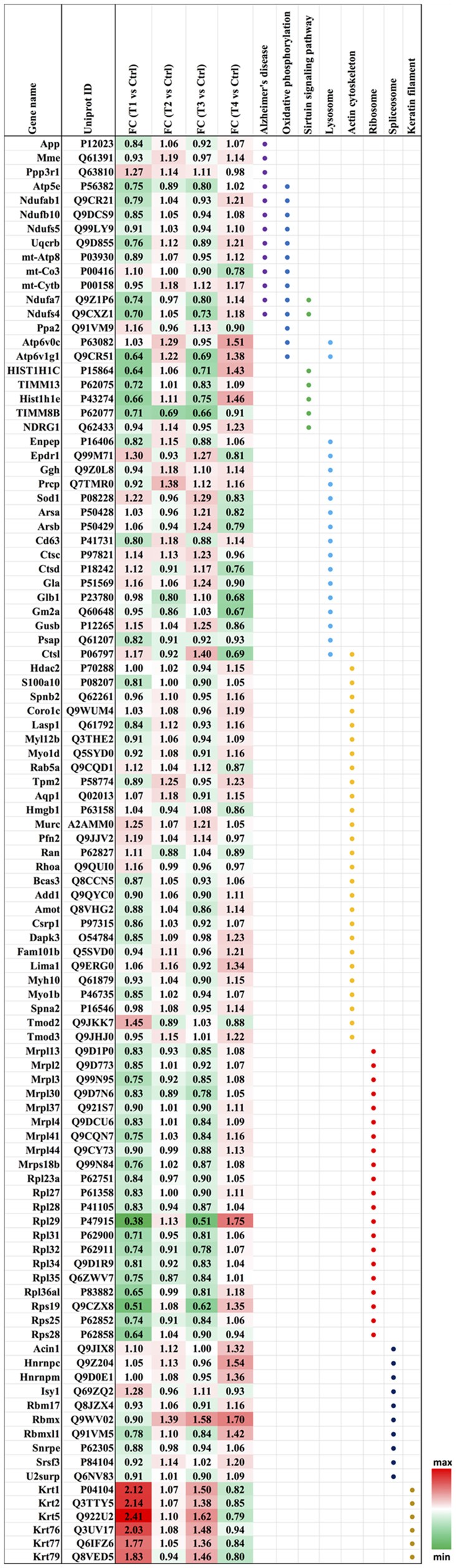
Heatmap of the regulated proteins in pathways related to AD. Red and green colors indicate relative increase or decrease in protein abundance, respectively.

Biochemical pathway analysis was used to reveal the molecular mechanisms, as well as the disease and biological processes affected in cells treated with Aβ. To achieve this, IPA was performed on the differentially expressed proteins identified in each comparison tests. The canonical pathway analysis (Figure 4B) in four treatments revealed that transcription signaling pathways particularly mediated through Elf2, elf4 and p70S6K were differentially affected. Oxidative phosphorylation and mitochondrial dysfunction linked pathways were also affected at both the Aβ concentrations with respect to time (6 and 24 h). Protein kinase A and Sirtuin linked signaling were similarly affected with maximum effect on differentially expressed proteins observed at 6 h time-point. Autophagy was an interesting pathway that was primarily induced at 25 μM concentration suggesting the potential toxicity effects. The network of interacting proteins found to be differentially expressed among all groups based on ANOVA (Figure 4C) was generated using the Cytoscape network environment with the StringDB plugin. Several enriched KEGG pathways are indicated on the network diagram, which complemented and reinforced the results of the analysis using IPA based on pairwise comparison. Apart from similar pathways identified in IPA (ribosome, sirtuin signaling and oxidative phosphorylation), other pathways such as spliceosome, lysosome, actin cytoskeleton and keratin filaments were uniquely enriched in STRING analysis. Differentially expressed proteins in these pathways were highlighted visually and shown in (Figure 5). The expression regulation shown in Figure 5 reinforced the results of the IPA analysis based on pairwise comparison. For instance, regulation of proteins in the ribosome dysfunction pathway was prominent in 5 μm_6 h (Figures 4B, 5) and proteins in sirtuin signaling pathway were significantly regulated in all treatment groups (Figures 4B, 5). All these results revealed the consistency within two different approaches based on pairwise comparison and ANOVA analysis. Effects on these proteins and pathways reflected that Aβ exhibits a differential effect with respect to both the concentration and time, but the highest disparity is observed with respect to timing rather than the indicated concentrations (5 and 25 μM).

### Ribosomal Proteins Were Negatively Affected Early Upon Amyloid β Treatment

We identified a significant down-regulation of several cytoplasmic as well as mitochondrial ribosomal proteins (21 proteins) in the early stages of the toxicity induced by Aβ treatment. Down-regulation of ribosomal proteins was identified at both 5 and 25 μM concentrations and in particular a strong effect on the protein levels was observed for Rpl29 (0.38–0.51-fold) and Rps19 (0.51–0.62-fold) proteins. Interestingly, the down-regulation of ribosomal proteins was alleviated when the photoreceptor cells were incubated with Aβ (5 μM) for longer time (24 h) with both Rpl29 and Rps19 demonstrating an opposite trend. These time dependent effects of Aβ were also observed in the cells treated with Aβ (25 μM) treated for 24 h. Both Rpl29 and Rps19 along with Rpl36a1 demonstrated a strikingly opposite effect with 1.75–1.2-fold protein upregulation (Figure 5). These results suggested that protein synthesis and elongation pathways were differentially affected by Aβ in the photoreceptor cells and the effects were dependent on both concentration of Aβ as well as duration of treatment.

### Initial Down-Regulation of Oxidative Phosphorylation Associated Pathways

In our dataset we identified the subset of proteins associated with the oxidative phosphorylation and mitochondrial dysfunction, which were differentially perturbed upon Aβ treatment. Interestingly, most of the proteins identified in this category (13 proteins) were negatively affected at both the 5 and 25 μM concentrations at 6 h time-point which evidently indicated that this pathway was affected early upon exposure to Aβ. Amongst these proteins Ndufs4 (0.7-fold) and Atp6v1g1 (0.65-fold) were prominently down regulated. The downward trend was blocked when cells were treated with lower concentrations (5 μM) of Aβ for 24 h. A prominent modulatory effect was observed in the photoreceptor cells treated with Aβ at higher concentrations (25 μM) for 24 h with most of the proteins demonstrating increased levels. The two most down-regulated proteins Ndufs4 and Atp6v1g1 (5 μM, 6 h) showed an increase of 1.18 and 1.38-fold, respectively when treated with 25 μM Aβ for 24 h. Atp6v0c exhibited an increase of 1.51-fold but it is important to highlight that some of the proteins such as mt-Co3 and Ppa2 that largely remained unchanged at lower concentrations of Aβ (5 μM, 6 h) were negatively affected (0.78–0.9-fold, respectively) at high concentration/longer time (25 μM, 24 h) treatments. Incidentally, several of the protein expression changes caused by Aβ treatment paradigms coincided with the way these pathways are affected in AD (10 proteins; Ke et al., 2012; Mangieri et al., 2014). APP expression was not altered in any of the treatment groups suggesting that although these changes are induced by Aβ but were not mediated through potential changes in parent APP protein. Interestingly, two of the affected proteins linked with oxidative phosphorylation Ndufa7 and Ndufs4 were also associated with Sirtuin signaling. Several other proteins of Sirtuin signaling (total seven proteins) were identified in the study and most proteins associated with this pathway showed a similar trend with protein down-regulation at 6 h and up-regulation at 24 h time points.

### Spliceosome Associated Pathways Are Induced by Aβ Upon Long Term Treatment

Several components of the spliceosome were identified (10 proteins) as differentially expressed across these four Aβ treatment groups suggesting that spliceosome and the mRNA processing were affected by Aβ. A closer look indicated that several proteins were either downregulated or unchanged at 6 h time-point at both the concentrations. However, at 24 h, several proteins demonstrated upregulated expression and the extent of protein upregulation was more pronounced when the photoreceptor cells were treated with higher concentrations of Aβ (25 μM). The most noticeable indication of protein upregulation with respect to time was evident for Rbmxl1 (0.78–1.1-fold change for 5 μM and 0.84–1.42-fold change 25 μM). In contrast, Isy1 exhibited an opposite effect with protein downregulation across both these concentrations when incubated with Aβ for 24 h. RNA binding motif protein Rbmx was unchanged at 5 μM (6 h) but was significantly modulated with increase in incubation time at both these concentrations.

### Reorganization of Cytoskeleton Networks and Effects on Cellular Viability

**Figure 6:**
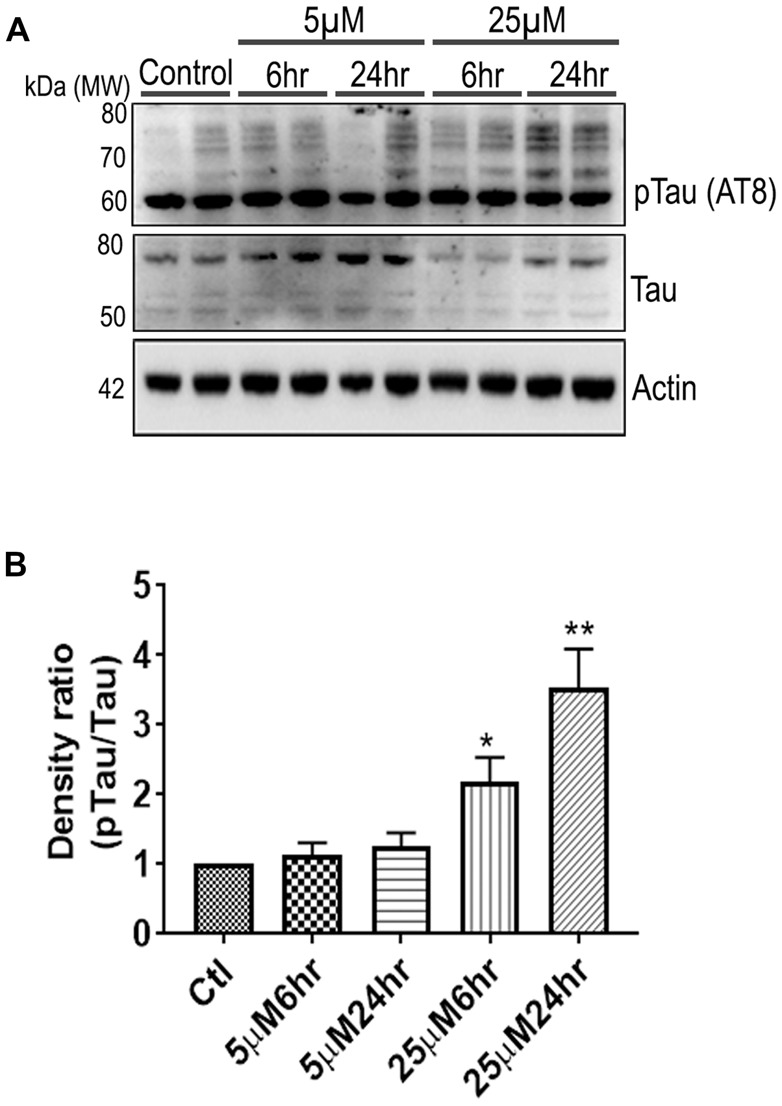
661 W Cells (1 × 10^6^) were cultured in plates and treated with 5 μM and 25 μM Aβ concentrations and harvested after 6 h and 24 h respectively. Cells were washed with ice cold 1× PBS, homogenized and subjected to **(A)** western blotting and probed with indicated antibodies- pTau Ser202/Thr205 (1:1,000), Tau (Tau46, 1:1,000) Anti-beta Actin (1:10,000). Blots were subjected to chemiluminescent substrate detection for HRP linked secondary antibody and **(B)** quantified by densitometric analysis (**p < 0.05, *p < 0.01).

**Figure 7:**
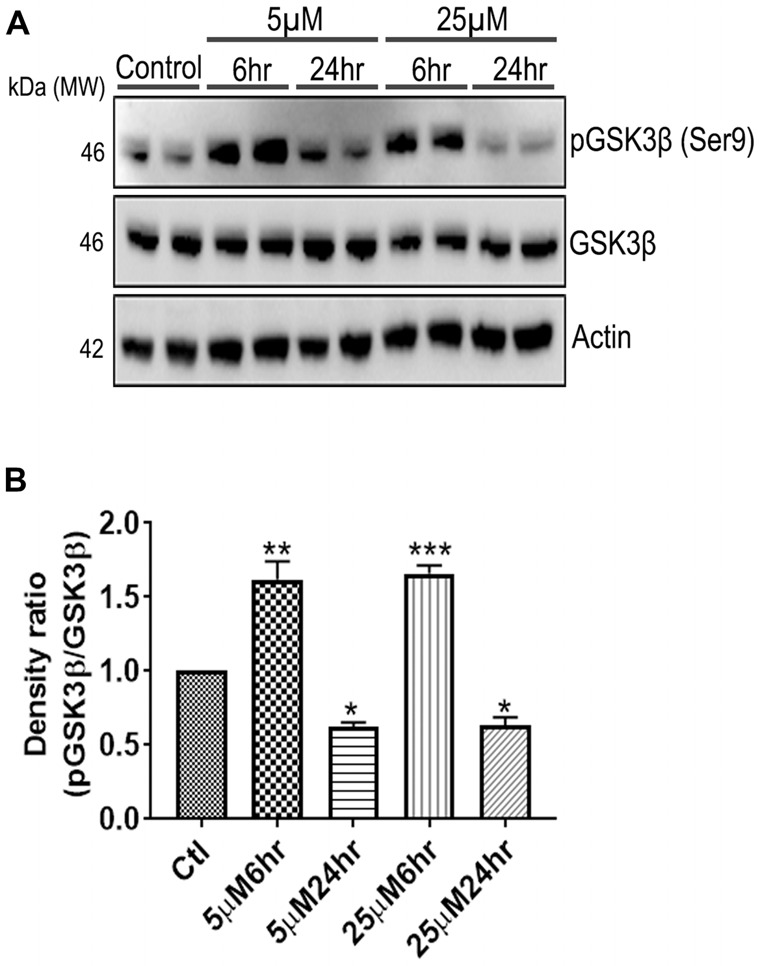
661 W Cells (1 × 10^6^) were cultured in plates and treated with 5 μM and 25 μM Aβ concentrations and harvested after 6 h and 24 h respectively. Cells were washed with ice cold 1× PBS, homogenized and subjected to **(A)** western blotting and probed with indicated antibodies- pGSK3β Ser9 (1:1,000), GSK3β (1:1,000), Anti-beta Actin (1:10,000). Blots were subjected to chemiluminescent substrate detection for HRP linked secondary antibody and **(B)** quantified by densitometric analysis (***p < 0.001, **p < 0.05, *p < 0.01).

**Figure 8:**
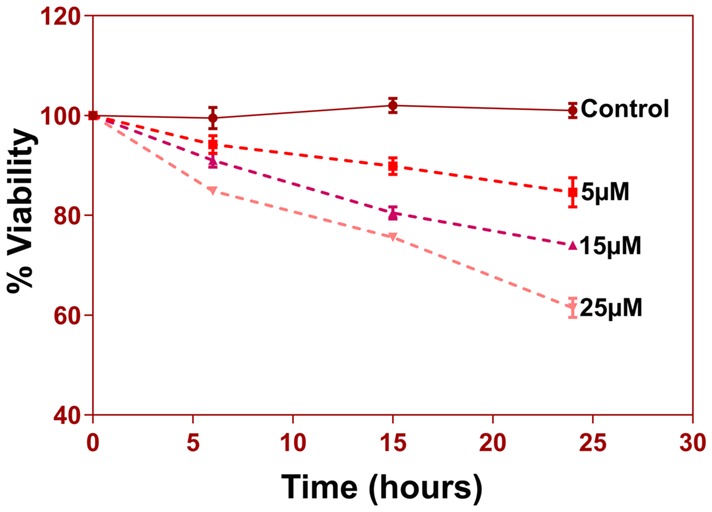
5 × 10^3^ cells per well were seeded in 96-well plate in triplicates for different groups. Cells were treated with 5, 15 and 25 uM of Aβ (1–42) for three different time durations 6 h, 15 h, and 24 h. At the end of respective treatments media was discarded and MTT reagent [3-(4,5-Dimethyl-2-thiazolyl)-2, 5-diphenyl-2H-tetrazolium bromide, M5655, Sigma Aldrich] was added to the wells at a final concentration of 0.5 mg/ml and incubated in a 37°C, CO_2_ incubator for 4 h for reduction of MTT to purple Formazan crystals. These crystals were dissolved in 200 μl of DMSO and incubated for 15–30 min with gentle shaking. The absorbance of the solution was read at 570 nm and percent viability was calculated.

Another prominent biochemical module that was differentially affected with respect to Aβ treatment was actin cytoskeleton (28 proteins) and keratin filament expression (six proteins). Network analysis of these proteins reflected that the pathways associated with mechanical support to cellular shape and internal organizations were affected. Many proteins in this network node were typically either unaffected or marginally downregulated in 6 h treatment for both 5 and 25 μM. At longer time point of 24 h treatment group, the proteins were again either unaltered or demonstrated slightly elevated levels. While, a greater proportion of proteins were up-regulated in the photoreceptor cells treated with higher concentrations (25 μM) for longer time (24 h). Interestingly, tropomodulin 2 showed a reverse trend with significant upregulation at 5 μM, 6 h (1.45-fold) and then a subtle downward trend at 24 incubation time. Murc and Pfn2 were two other proteins that were induced at 5 μM, 6 h but were not altered in other experimental paradigms compared to control cells. Keratin filament associated proteins intriguingly demonstrated a reverse trend with consistent protein upregulation in early stages of the Aβ treatment (6 h) at both 5 and 25 μM concentrations. The upregulation trend indeed was more prominent at 5 μM concentration. In contrast, the keratin associated proteins showed no expression changes when incubated for 24 h with 5 μM Aβ and a slight downregulation across the spectrum of these proteins was evident when the photoreceptor cells were incubated with the 25 μM peptide for 24 h. Tau is major cytoskeleton protein that undergoes increased phosphorylation under AD conditions (Bloom, 2014). We sought to investigate Tau expression and phosphorylation changes in the photoreceptor cells in response to Aβ treatment. Our results indicated enhanced phosphorylation of Tau in response to Aβ treatment across both the concentrations. Tau phosphorylation was particularly elevated when the cells were treated with higher concentrations of Aβ (25 μM) at both the time-points (Figure 6). No significant Tau protein expression changes were observed in any of the groups. Parallel to Tau hyperphosphorylation (Ser202/Thr205), we also observed increased GSK3β phosphorylation in response to Aβ treatment. GSK3β dysregulation has strongly been implicated in AD pathogenesis and promotes cellular senescence (Reddy, 2013). The Ser/Thr kinase is shown to play a role in both Aβ production and its toxic effects leading to neuronal death (Hernandez et al., 2013). We observed initial upregulation of GSK3β phosphorylation at both the concentrations (6 h) which was found to be subsequently decreased at 24 h (Figure 7). We sought to investigate whether Aβ treatment led to cell survival changes in photoreceptor cells at different concentrations of the peptide (5, 15 and 25 μM) over a period of time (0–24 h). Our findings suggest that cell viability was inversely related to the Aβ dose and time of treatment (Figure 8). MTT data at least partially reflects the reduced mitochondrial function that was also identified in mass spectrometry data upon Aβ treatment in 661 W cells. Together, these results indicate that toxic effects of Aβ might be linked with impaired cytoskeleton and underlying aberrations with filamentous architecture and that these effects correlated with increased Tau phosphorylation, GSK3β dysregulation and reduced cell viability.

## Discussion

Aβ has been shown to induce toxicity effects in the retina with ageing and in various disease conditions (Gupta et al., 2014, 2016a,b; Mirzaei et al., 2017a). This study established that Aβ 1–42 fragment induced significant perturbations in various biochemical networks in the photoreceptor neurons in culture conditions. We identified that Aβ 1–42 fragment which is known to exhibit toxic effects in CNS induced significant perturbations in the expression of various proteins in the 661 W photoreceptor cells. The effects of Aβ were highly dependent on the peptide concentration and the duration of exposure. Analysis of the proteomics changes indicated that timing of exposure had a more profound effect in inducing overall proteome changes in photoreceptor cells rather than the peptide concentration by itself. The major biochemical pathways that were affected in response to Aβ treatment were ribosomal protein synthesis, oxidative phosphorylation, cytoskeleton associated proteins, lysosomal regulation and mRNA processing machinery of spliceosomes.

Ribosomal dysfunction has been demarcated as one of the initial processes in AD pathogenesis (Ding et al., 2005). Several genes associated with protein initiation, translation and elongation have been shown to be abnormally affected in the ribosomes (Hernandez-Ortega et al., 2016). Ribosomes are the site of initiation of protein synthesis and its elongation and we identified that both mitochondrial and cytoplasmic ribosomal proteins were affected. Indeed mitochondrial dysfunction has consistently been reported in AD associated neurodegeneration (Lunnon et al., 2017) and Aβ has been shown to localize at the mitochondrial membrane, which may be one of the sites of Aβ toxicity effects, potentially through its inhibition of pre-protein maturation (Spuch et al., 2012; Mossmann et al., 2014). Data analysis signified downregulation of ribosomal proteins in the early stages of Aβ treatment (6 h) that rebounded to its initial levels when incubated for longer period of time (24 h) particularly at the higher concentrations of Aβ (25 μM). These differential effects of Aβ may be attributed to recovery of the cells from stress induced by the peptide during initial phases of incubation and subsequent bouncing back of the biochemical processes. Increased clearance of Aβ from the cells either due to its aggregation, internalization in the lysosomes or proteolytic digestion may also lead to reduced effects of Aβ over a period of time. Indeed, in this respect, we also observed concomitant upregulation of lysosomal proteins in the early phases of Aβ treatment (6 h) at both the concentrations that subsided over a period of time and demonstrated even a subtle downregulation of lysosomal proteins (24 h). Autophagy-lysosomal system is believed to be one of the key mechanisms to eliminate Aβ and its impaired status in AD is an important focus of drug development (Tarasoff-Conway et al., 2015). Autophagy of misfolded and aggregated proteins through lysosomal-endosomal system has been shown to protect against retinal neuro-inflammation and degeneration associated with drusen in macular degeneration (Buschini et al., 2011; Sinha et al., 2016). Aβ assemblies have been extensively reported in drusen deposits in the retina that might be involved in chronic complement activation and photoreceptor degeneration (Johnson et al., 2002; Schuman et al., 2009).

Aβ along with Tau protein together induce a decline in oxidative phosphorylation in animal models of AD (Rhein et al., 2009). Altered metabolism of APP has been shown to associate with mitochondrial dysfunction in primary cortical neurons in AD and in other disease conditions associated with anomalous Aβ processing such as in down’s syndrome (Busciglio et al., 2002; Casley et al., 2002; Mao and Reddy, 2011). We identified that Aβ treatment induced a downregulation of several proteins associated with mitochondrial regulation and oxidative phosphorylation (Figure 5). Interestingly, the differential regulation of proteins was more evident at 6 h at either concentration compared to the 24 h suggesting that this key cellular process is affected early upon exposure to Aβ and might comprise an initial event of the peptide toxicity. The protein changes seemingly recovered at the longer time point of 24 h with some of the proteins showing significant upregulation. Remarkably, a substantial overlap of several of the oxidative phosphorylation proteins was observed with the proteins that are affected in AD (Figure 5). APP expression remained largely unaltered in various experimental conditions suggesting that observed proteomics variations were not indirectly mediated through potential effects of Aβ on endogenous APP expression. Parallel to the mitochondrial machinery, sirtuin signaling demonstrated a significant downward trend in the initial phases of Aβ exposure that either returned back to initial levels or was upregulated at 24 h (Figure 5). Tau protein accumulation has been shown to be inversely associated with Sirtuin signaling in AD (Julien et al., 2009). This pathway is implicated in protecting the cells against Aβ toxic effects by its inhibitory effects on NFκB signaling (Chen et al., 2005). SIRT1 was shown to negatively regulate the mTOR signaling (Ghosh et al., 2010) and this study revealed an inverse correlation between differential regulation of Sirtuin and mTOR signaling at higher concentrations of 25 μM peptide in the photoreceptor cells (Figure 4B). More recently, SIRT3, a key component of sirtuin signaling has been shown to impart neuroprotection against light toxicity in the retina and its suppression led to increased ROS production in 661 W cells (Ban et al., 2017).

Proteomics studies have previously implicated RNA splicing defects in AD (Ke et al., 2012). Nuzzo et al. (2017) recently identified spliceosome impairment as a prominent pathway that was affected in LAN5 neuroblastoma cells upon Aβ treatment (Nuzzo et al., 2017). Mutations in splicing factors Rp9, DHX38 and several snRNP proteins such as PRPF3 have independently been shown to lead to photoreceptor degeneration and form aggregates in 661 W photoreceptor cells (Comitato et al., 2007; Lv et al., 2017; Ruzickova and Stanek, 2017). Aβ treatment of 661 W photoreceptor cells in this study revealed subtle downregulation or no change in the initial stages of peptide treatment (6 h), however several proteins were upregulated 24 h following treatment with higher Aβ concentrations (25 μM). These findings supported the premise that alterations in RNA splicing may be induced at a relatively prolonged exposure to Aβ. It is important to highlight that there was also a recovery of ribosomal protein levels at this time-point (Figure 5), which could potentially underlie an increased synthesis of spliceosome associated proteins in the ribonucleoprotein particle (RNP) assemblies.

Finally, another major biochemical module that was identified to be differentially regulated was the actin cytoskeleton and keratin filament associated protein network. Cytoskeleton associated proteins demonstrated a slight upregulation when exposed to peptide concentration of 25 μM for 24 h (Figure 5). Under normal conditions there is a constant turnover between the globular and filamentous cytoskeleton proteins for the proper cell functioning, however Aβ might affect the assembly/disassembly of cytoskeleton proteins and impair cellular adaptability (Cardenas et al., 2012). Supporting our observations, Aβ treatment has previously been shown to induce dysregulation of actin cytoskeleton proteins in the neurons by 12–24 h following treatment (Deshpande et al., 2006; Bamburg and Bloom, 2009). In the retina, sub-retinally injected Aβ 1–42 was shown to induce dis-organization of actin cytoskeleton in the RPE cells leading to subsequent photoreceptor loss (Bruban et al., 2009). An interesting observation was the remarkable enrichment of the keratin filament associated proteins by Aβ at 6 h time point that basically bounced back to normal levels at 24 h at lower concentrations of Aβ (5 μM). This might suggest that keratin proteins are potentially impacted early in response to Aβ exposure. Keratin 8 has been shown to protect the RPE cells against oxidative stress induced injury and keratin 9 was even suggested to be one of the biomarkers of AD (Richens et al., 2016), although it is plausible that the changes that we identified in this study are 661 W cell specific owing to their retinal origin. Tau is another major cytoskeleton associated protein that undergoes hyperphosphorylation in AD and forms intracellular tangles. Increased Tau phosphorylation observed in this study in response to high Aβ concentrations for longer period corresponded with GSK3β activation (Figures 7, 8). Conversely, at low concentrations of Aβ (5 μM, 6 h), GSK3β was inactivated and accordingly no Tau phosphorylation changes were observed. The two proteins have been suggested to play key converging roles in AD pathogenesis (Hernandez et al., 2013) with GSK3β inducing Tau phosphorylation as a substrate in cells (Avila et al., 2012). Increased Tau phosphorylation (25 μM, 24 h) interestingly, also corresponded with upregulation of expression of several other cytoskeleton associated proteins (Figure 5) observed at higher concentrations of Aβ (25 μM, 24 h). GSK3β inhibition in response to 6 h Aβ treatment (5 and 25 μM) observed here, may be an early event when the cells are trying to counter the neurotoxic effects of the peptide by activating neuroprotective pathways. Aβ-induced neurotoxicity has previously been shown to be reduced upon GSK3β inhibition (Koh et al., 2008). Following 24 h of incubation, GSK3β was observed to be in an activated state across both Aβ concentrations. It is possible that these time dependent effects of Aβ on GSK3β activity is a 661 W photoreceptor cell specific response, and future *in vivo* studies will unravel the molecular basis of these actions in comparison with other neuronal cells. Changes in GSK3β activation state in response to Aβ treatment match the trends identified in our other results where we observed that proteome profile of 661 W photoreceptor cells was similarly affected when treated for 6 h at either 5 or 25 μM Aβ concentrations. A distinct pattern of protein changes was also evident within the cells that were subjected to 24 h treatment (5 and 25 μM).

We selected a low concentration of 5 μM and a moderately higher concentration of 25 μM to study the effects of the Aβ peptide on the photoreceptor cells. At 25 μM, we observed significant enrichment of the autophagy canonical pathway in the cells (Figure 4B) suggesting that treating the cells with a much higher concentration could have induced rampant cell death and confound the protein expression changes within the cells. This study revealed initial proteomics changes without the confounding effects of the widespread apoptosis and cell-death. Cell survival assays at different concentrations of Aβ and incubation timings revealed an inverse relationship with cellular viability. These studies further demonstrated that molecular degenerative changes delineated in this study, in response to Aβ treatment play a key role in photoreceptor cell survival under culture conditions. Further, other fragments of Aβ, such as Aβ 1–40 and Aβ 1–16 are known and exhibit distinct biological functions. Pharmacological treatments with these fragments may differentially affect various biochemical pathways in these cells. It is however pertinent to mention that Aβ 1–42 is considered highly toxic compared to other smaller fragments, is generally present at a much higher concentration compared to Aβ 1–40 and is also more prone to aggregate formation (Suh and Checler, 2002; De Strooper, 2010; Gupta et al., 2014).

While autophagy pathways were observed to be upregulated, we could not identify caspase activation in response to Aβ treatment in 661 W photoreceptor cells. Caspase activation has previously been reported in other cell lines upon Aβ treatment and in AD brains (Dickson, 2004; Sharoar et al., 2014). These differences could be attributed to possibly lower expression of these proteins in 661 W cells or different mechanisms of Aβ toxicity in retinal cells. The extensive alterations in proteome profile caused by Aβ in the photoreceptor cells is important, although the exact physiological functions of APP or disease specific roles of Aβ in the outer retina are not known. This can be studied by performing intravitreal injections of Aβ 1–42 in the animals and determining molecular changes in the retina. It is possible that in the retina many of the implication of the peptide related to its toxicity effects are sub-clinical and not easily identifiable with current imaging tools. We provide these data as a resource for future investigations in animal and human studies, which are needed to conclusively establish the biochemical effects of Aβ in the retina, which can then be used for therapeutic targeting. Follow up investigations on the mechanisms of action of the Aβ peptide will determine sequelae of biochemical events that are triggered in response to the peptide in the photoreceptors and other retinal neurons such as in retinal ganglion cells that are particularly affected in glaucoma and AD pathology (Gupta et al., 2014, 2016a,b).

## Author Contributions

MM and VG conceived and designed the study. LD, KP, CJ, VKG, NC, YD, AA, KK, DP, JW, MM and VKG performed the experiments, analyzed and interpreted the data. MM and VKG wrote the manuscript. SG, GS and PH critically revised the manuscript. All authors read and approved the final manuscript.

## Conflict of Interest Statement

The authors declare that the research was conducted in the absence of any commercial or financial relationships that could be construed as a potential conflict of interest.

## Footnotes

^1^http://www.ingenuity.com/index.html

^2^http://apps.cytoscape.org/apps/stringapp
