# Supplementary material for: Amyloid β Induces Early Changes in the Ribosomal Machinery, Cytoskeletal Organization and Oxidative Phosphorylation in Retinal Photoreceptor Cells
Source: Front Mol Neurosci. 2019 Feb 22;12:24. doi: 10.3389/fnmol.2019.00024 (PMC6395395; doi:10.3389/fnmol.2019.00024)
Supplement: Supplementary file 4 [file Table_4.DOCX]

**Supplementary information**

**Supplementary Dataset 1** | The combined set of identified proteins from two TMT experiments performed on retinal photoreceptor cells with Aβ1-42 fragment treatments and control.

**Supplementary Dataset 2** | The combined four sets of differentially expressed proteins obtained from a student t-test comparison between the specific treatment of Aβ1-42 fragment on retinal photoreceptor cells and the control.

**Supplementary Dataset 3** | Quantitative proteome profiling reveals different protein expressions in four specific treatments on retinal photoreceptor cells with Aβ1-42 fragments and the control using ANOVA analysis.
